# Influenza-Associated Disseminated Aspergillosis in a 9-Year-Old Girl Requiring ECMO Support

**DOI:** 10.3390/jof7090726

**Published:** 2021-09-05

**Authors:** Natalia Mendoza-Palomar, Susana Melendo-Pérez, Joan Balcells, Jaume Izquierdo-Blasco, Maria Teresa Martín-Gómez, Monica Velasco-Nuño, Jacques G. Rivière, Pere Soler-Palacin

**Affiliations:** 1 Paediatric Infectious Diseases and Immunodeficiencies Unit, Hospital Universitari Vall d’Hebron, 08035 Barcelona, Spain; smelendo@vhebron.net (S.M.-P.); jriviere@vhebron.net (J.G.R.); psoler@vhebron.net (P.S.-P.); 2 Infection in the Immunosuppressed Paediatric Patient Research Group, Vall d’Hebron Research Institute, 08035 Barcelona, Spain; 3 Paediatric Intensive Care Unit, Hospital Universitari Vall d’Hebron, 08035 Barcelona, Spain; jbalcell@vhebron.net (J.B.); jizquierdo@vhebron.net (J.I.-B.); 4 Clinical Research/Innovation in Pneumonia and Sepsis (CRIPS) Research Group, Vall d’Hebron Research Institute, 08035 Barcelona, Spain; 5 Microbiology Department, Hospital Universitari Vall d’Hebron, 08035 Barcelona, Spain; mtmartin@vhebron.net; 6 Microbiology Research Group, Vall d’Hebron Research Institute, 08035 Barcelona, Spain; 7 Nuclear Medicine Department, Hospital Universitari Vall d’Hebron, 08035 Barcelona, Spain; mvelasco@vhebron.net; 8 Molecular Medical Imaging Research Group, Vall d’Hebron Research Institute, 08035 Barcelona, Spain

**Keywords:** invasive pulmonary aspergillosis, neuroaspergillosis, human influenza, isavuconazole, children

## Abstract

A previously healthy 9-year-old girl developed fulminant myocarditis due to severe influenza A infection complicated with methicillin-resistant *Staphylococcus aureus* pneumonia, requiring extracorporeal membrane oxygenation (ECMO) support. Twelve days after admission, *Aspergillus fumigatus* was isolated in tracheal aspirate, and 12 h later she suddenly developed anisocoria. Computed tomography (CT) of the head showed fungal brain lesions. Urgent decompressive craniectomy with lesion drainage was performed; histopathology found hyphae in surgical samples, culture-positive for *Aspergillus fumigatus* (susceptible to azoles, echinocandins, and amphotericin B). Extension workup showed disseminated aspergillosis. After multiple surgeries and combined antifungal therapy (isavuconazole plus liposomal amphotericin B), her clinical course was favorable. Isavuconazole therapeutic drug monitoring was performed weekly. Extensive immunological study ruled out primary immunodeficiencies. Fluorine-18 fluorodeoxyglucose positron emission tomography/CT (^18^F-FDG PET/CT) follow-up showed a gradual decrease in fungal lesions. Influenza-associated pulmonary aspergillosis is well-recognized in critically ill adult patients, but pediatric data are scant. Clinical features described in adults concur with those of our case. Isavuconazole, an off-label drug in children, was chosen because our patient had severe renal failure. To conclude, influenza-associated pulmonary aspergillosis is uncommon in children admitted to intensive care for severe influenza, but pediatricians should be highly aware of this condition to enable prompt diagnosis and treatment.

## 1. Case Description

In February 2019, a previously healthy 9-year-old girl consulted in the emergency department with a 24-h history of runny nose, sore throat, cough, fever (39 °C), and progressive shortness of breath. On examination, she showed respiratory insufficiency and hemodynamic shock requiring intubation and pediatric intensive care unit (ICU) admission for supportive treatment. Further workup found severe leucopenia (500 leucocytes/µL, 100 neutrophils/µL), C-reactive protein 10 mg/dL, bilateral opacities on chest X-ray, and left ventricular dysfunction, consistent with fulminant myocarditis. She required venoarterial extracorporeal membrane oxygenation (ECMO) support and renal replacement therapy. Percutaneous atrioseptostomy was performed for left ventricular unloading. Based on the patient’s condition, severe neutropenia, and suspected myocarditis, empirical treatment with cefepime, azithromycin, foscarnet, and immunoglobulins was started, together with vancomycin as systematic ECMO prophylaxis. Nasopharyngeal swab testing was positive for influenza A H3N2, and oseltamivir was added. Inflammatory infiltrates on endomyocardial biopsy confirmed myocarditis; hence, methylprednisolone (2 mg/kg/day) was started. Blood and tracheal aspirate culture showed methicillin-resistant *Staphylococcus aureus* (MRSA), whereas the workup was negative for viruses (in both blood and endomyocardial biopsy), which led to foscarnet withdrawal.

**Figure 1:**
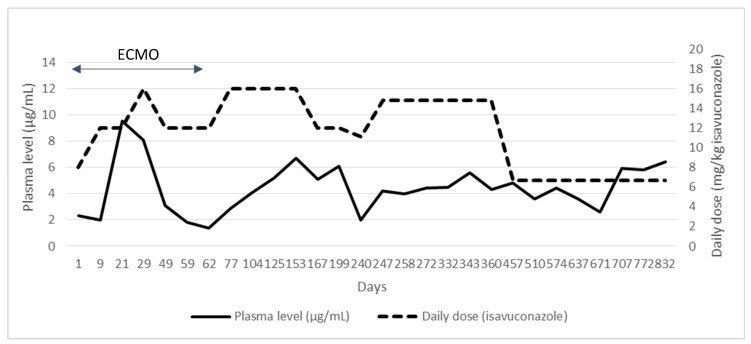
Plasma levels and isavuconazole dosage during the patient’s clinical evolution.

Initially, the patient remained stable on ECMO, and neutropenia recovered 4 days after admission. Methylprednisolone was tapered after 6 days. Blood, tracheal aspirate, and urine cultures were performed every other day, with negative results until day +12, when *Aspergillus fumigatus* was isolated from tracheal aspirate, with no clinical correlation. Twelve hours later, she suddenly developed anisocoria; computed tomography (CT) of the head showed fungal brain lesions. Decompressive craniectomy with lesion drainage was performed immediately with the patient on ECMO. The extension workup showed lesions suggestive of invasive fungal disease in multiple sites, including the central nervous system (CNS), paranasal sinuses, left eye, lungs, heart, spleen, bone, and soft tissues. *A. fumigatus* was again detected on tracheal and bronchial aspirates, and galactomannan antigen was positive in serum and bronchoalveolar lavage (4.1 and 3.7, respectively). Histopathological examination found hyphae in CNS surgical samples, later testing positive for *A. fumigatus* (susceptible to azoles, echinocandins, and amphotericin B). She was started on a 3-month combined antifungal therapy course with isavuconazole and liposomal amphotericin B (L-AmB). Although isavuconazole use is off-label in children, it was chosen due to renal failure requiring continuous renal replacement therapy. Isavuconazole therapeutic drug monitoring (TDM) was performed weekly, considering 2.5–5 µg/mL the desired therapeutic range. In total, 48% of plasma drug levels were outside the range, leading to dose adjustments (Figure 1).

A detailed clinical history performed by an expert inmunologist found no data suggestive of prior neutropenia nor other immune defects. Immunoglobulin values were normal at admission and subsequent analysis of lymphocyte subsets showed no abnormalities: naive lymphocyte number and function were normal, as was the neutrophil burst test. Genetic testing included a specific inborn errors of immunity gene panel and whole-exome sequencing. No known pathogenic mutations were found, with special focus on those related with severe influenza.

**Figure 2:**
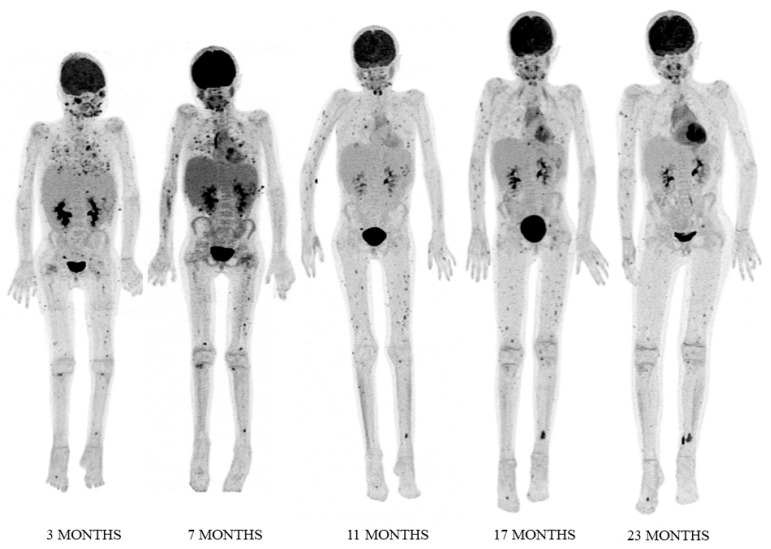
Fluorine-18 fluorodeoxyglucose positron emission tomography **(**^18^F-FDG PET) maximum intensity projection (MIP) images at 3, 7, 11, 17, and 23 months after the diagnosis of *Aspergillus* infection. PET studies showed resolution or reduction of FDG uptake in most fungal lesions located in the paranasal sinuses, left eye, lungs, spleen, bone, and soft tissues (see also Figure 3). A soft tissue lesion in the left leg was the only one showing increased uptake (see also Figure 3; image F).

**Figure 3:**
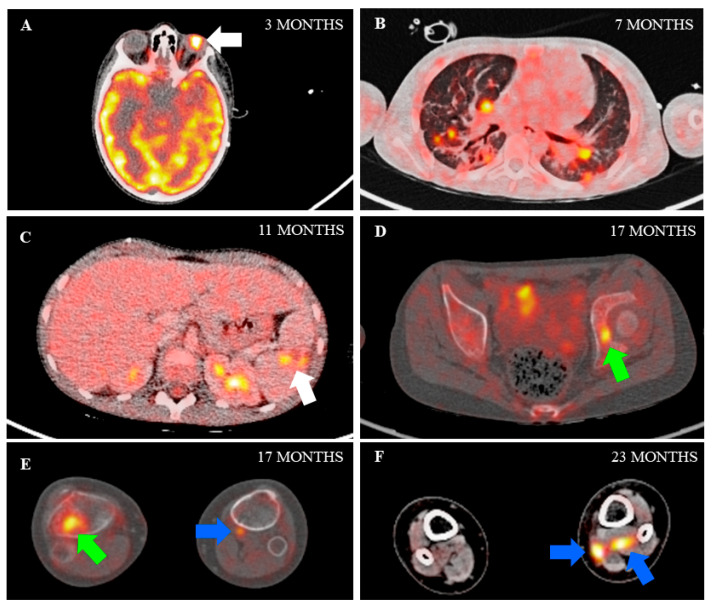
Fused fluorine-18 fluorodeoxyglucose positron emission tomography/CT (^18^F-FDG PET/CT) images at various time points show pathological FDG uptake in the left eye (image (**A**); arrow), lungs (image (**B**)), spleen (image (**C**); arrow), bone (images (**D**,**E**); green arrows) and muscles (images (**E**,**F**); blue arrows).

The patient’s clinical status was favourable after 73 days of ECMO. L-AmB was withdrawn on day 90. Serum galactomannan antigen tested negative after 50 days of treatment, but fluorine-18 fluorodeoxyglucose positron emission tomography/CT (^18^F-FDG PET/CT) follow-up revealed persistent activity of nearly all fungal lesions, including the left eye and spleen (Figure 2 and Figure 3A,C). As the left eye presented complete amaurosis, it was enucleated, and splenectomy was performed some months later due to development of new lesions and persistent inflammation (moderate acute phase reactant elevation, anemia, failure to thrive). Histopathological examination found hyphae suggestive of *Aspergillus* spp. in both tissues, but fungal culture was negative. Panfungal polymerase chain reaction analysis was positive for *A. fumigatus* only in eye samples.

^18^F-FDG PET/CT imaging after splenectomy showed resolution of infection in the paranasal sinuses, skin, and lungs, and improvements in most fungal lesions in bone and soft tissues. However, a soft tissue infection in the left leg showed abnormal uptake (Figure 2 and Figure 3).

At the time of writing, the patient is clinically stable under oral isavuconazole treatment. She has sequelae of spastic paraparesis and episodic seizures with normal cognition, which are slowly improving with rehabilitation. She has resumed schooling.

## 2. Discussion

Influenza-associated pulmonary aspergillosis (IAPA) has been well-described in adults, but to our knowledge, this is the first comprehensive description of IAPA in a child. The initial adult case reports date from 1952, with the number increasing after the influenza A H1N1 pandemics to include large multicenter cohort studies [1,2,3]. In a Spanish cohort of 2901 critical influenza patients, 35 (1.2%) IAPA cases were detected, with *Aspergillus* spp. being the fourth cause of superinfection [1]. A multicenter study from Belgium and the Netherlands reported a higher incidence of IAPA (83 cases in 423 critical influenza patients, 19%), establishing influenza as an independent risk factor for aspergillosis [4]. A large study including 19,697 ECMO patients also identified influenza as a risk factor for *Aspergillus* spp. colonization and co-infection [5]. Although the available studies have different designs and diagnostic criteria, the association between severe influenza and pulmonary aspergillosis in adults is convincing and has been the subject of a European Center for Disease Prevention and Control monography [6].

Nonetheless, pediatric data is scanty. Vallero et al. described a 10-year-old boy with acute promyelocytic leukemia and concomitant aspergillosis and influenza A H1N1, but the aspergillosis diagnosis preceded influenza detection by 9 days, thus questioning the IAPA diagnosis [7]. A necropsy series including 47 pediatric influenza cases identified 1 patient with *Aspergillus* spp. coinfection, detected in lung and brain abscesses by culture and immunohistochemical assay. Unfortunately, specific data about the patient were not provided [8].

Initially, IAPA was attributed to antibiotic use in complicated pneumonia. However, the association between influenza and aspergillosis is now thought to be mainly secondary to inflammatory changes produced by the virus itself, which causes cytokine release (mainly IL-10) and mucosal disruption, hampering the innate immune response to fungi. Other contributing factors include viral-induced neutropenia and the use of corticosteroids and devices [1,3,6].

Most adult cases have involved influenza A, but influenza B-related IAPA is also reported [2,9]. Neutropenia and immunosuppression are described as additional risk factors, although 28% to 54% of patients were previously healthy [2,4,10]. Of note, the time between ICU admission and IAPA detection was short (median 5 days (IQR 2–11.5 days)) [2] and the lung was predominantly affected, although cases of disseminated aspergillosis (some including the CNS) have been described [11]. This clinical picture, mainly reported in adults, is concordant with that of our clinical case. In addition to influenza, other factors could have contributed to her aspergillosis: broad-spectrum antibiotic treatment, self-limiting neutropenia, methylprednisolone treatment (although relatively short), and use of several medical devices (endotracheal tube, ECMO cannulas). It is likely that *A. fumigatus* was airborne transmitted during intubation and/or mechanical ventilation, leading to a primary pulmonary infection promptly followed by systemic dissemination.

Influenza is known to be fatal or near-fatal in some pediatric patients, especially those with predisposing conditions, such as acquired immunodeficiency or asthma [12]. Little is known regarding ostensibly immunocompetent patients, as cases of severe influenza in otherwise healthy patients are scarce. In the last decade, growing numbers of single-gene defects have been found that cause single Mendelian susceptibility to severe infection [13,14]. Severe influenza has been described in previously healthy individuals with underlying monogenic defects in interferon (I and/or III)-mediated immunity, such as GATA2, IRF7, IRF9, and TLR3. These defects have redundant pathways in some tissues and show incomplete penetrance, which may account for some “sporadic” cases [15]. Although IAPA is consistently described in adults, invasive aspergillosis should lead to extensive testing to rule out neutrophil defects such as chronic granulomatous disease, and STAT1GOF, CARD9 deficiency, leaky SCID, and GATA2, among other primary immune defects [16]. Most of these challenging cases can only be diagnosed with up-to-date gene panels or by whole-exome or whole-genome sequencing, highlighting the importance of genetic testing in severely ill patients.

Regarding treatment, isavuconazole use is off-label in children. Nonetheless, it was chosen as the cornerstone of the patient’s antifungal treatment because of concurrent severe renal failure that precluded the use of voriconazole or posaconazole due to potential cyclodextrin accumulation. Even though continuous replacement therapy can effectively remove cyclodextrin, thereby enabling voriconazole treatment, the use of this drug is still under study and has not been standarised for pediatric patients [17]. Isavuconazole lacks cyclodextrin and renal excretion of the drug is minimal, making it a useful alternative for patients with (even severe) renal failure, despite the limited experience in this setting [18]. Although dual therapy is not recommended in most guidelines, it was used initially due to the severity of invasive fungal infection with CNS involvement. Isavuconazole has demonstrated blood–brain barrier penetration in animal models, and its clinical effectiveness for CNS fungal infection has been shown retrospectively both in adults and in pediatric patients [19]. Although L-AmB penetration is lower, it has elicited favorable responses in patients with CNS aspergillosis, especially when combined with azoles [20,21]. Isavuconazole TDM is not recommended in adults, but dosing and pharmacokinetics in children are still under study; [22,23] hence, TDM was performed weekly. Therapeutic ranges were established based on data from adults and previous pediatric case reports [24,25].TDM led to dose changes on several occasions, mainly related to the patient’s clinical status (while on ECMO, doses were higher than when clinically stable). Before hospital discharge, administration was switched to oral route, maintaining therapeutic plasma levels with the same dose. Isavuconazole has been well-tolerated throughout.

Bimonthly ^18^F-FDG PET/CT follow-up of the fungal disease was used for overall assessment and to distinguish active from residual lesions. Recent studies by Ankrah et al. [26,27] have described ^18^F-FDG PET/CT monitoring of treatment response in patients with invasive fungal infection. As compared to standard CT imaging, ^18^F-FDG PET/CT provided information that changed the therapeutic decision in half the patients [26]. Later, these authors reported that ^18^F-FDG PET/CT enabled detection of a larger number of lesions than conventional anatomical imaging and differentiation between active and residual lesions [27]. Again, similar studies are lacking in pediatrics, but in our experience ^18^F-FDG PET/CT was useful for follow-up of disseminated aspergillosis, together with acute-phase reactant analysis and clinical criteria. Of note, serial serum galactomannan antigen determination was not a good predictor of the patient’s course after the acute phase of the infection, as it tested negative after seven weeks of treatment despite radiological and clinical evidence of persistent fungal infection.

IAPA has been described as an independent risk factor for mortality in severe influenza patients, with fatality rates as high as 67% [4,28]. Fortunately, our patient is alive and stable, but the infection led to considerable morbidity, with ongoing sequelae.

To conclude, IAPA is uncommon, but potentially life-threatening in children admitted to the ICU for severe influenza. Pediatricians should be highly aware of this condition to enable prompt diagnosis and treatment. 

## Data Availability

Not applicable.
